# Incremental Structure from Motion for Small-Scale Scenes Based on Auxiliary Calibration

**DOI:** 10.3390/s25020415

**Published:** 2025-01-12

**Authors:** Sixu Li, Jiatian Li, Tao Yang, Xiaohui A, Jiayin Liu

**Affiliations:** Faculty of Land and Resources Engineering, Kunming University of Science and Technology, Kunming 650093, China; lisixu@stu.kust.edu.cn (S.L.); 20222201093@stu.kust.edu.cn (T.Y.); 20211001001@stu.kust.edu.cn (X.A.); 20233101016@stu.kust.edu.cn (J.L.)

**Keywords:** auxiliary calibration, feature enhancement, multiple constraints, structure from motion (SfM)

## Abstract

Scarce feature points are a critical limitation affecting the accuracy and stability of incremental structure from motion (SfM) in small-scale scenes. In this paper, we propose an incremental SfM method for small-scale scenes, combined with an auxiliary calibration plate. This approach increases the number of feature points in sparse regions, and we randomly generate feature points within those areas. At the same time, we obtain a coarse matching set of feature points using pairwise polar geometric constraints. The positional results from the geometric constraints of the calibration plate are then used to filter out high-precision matching points, thereby improving the accuracy of the three-dimensional reconstruction. Experimental results demonstrate that the proposed method achieves superior reconstruction completeness and accuracy. In three real-world experiments, the average re-projection errors were 0.5245, 0.4151, and 0.4996 pixels, outperforming competing methods. This approach ensures robust pose estimation and facilitates precise 3D reconstructions.

## 1. Introduction

Structure from motion (SfM), a photogrammetric technique mainly aimed at reconstructing 3D structures from multiple 2D images, enables the sparse reconstruction of objects [1]. Current SfM algorithms can be broadly categorized into incremental methods [2,3,4] and global methods [5,6,7,8]. Incremental SfM begins with an optimal image pair for the initial reconstruction and progressively incorporates new images while performing bundle adjustments to ensure reconstruction accuracy. Global SfM, on the other hand, solves for all camera poses and 3D points simultaneously, avoiding accumulated errors but often exhibiting reduced robustness. To address these challenges, various approaches have been proposed. For example, Yang [9] utilizes geometric constraints such as parallelism and orthogonality in scenes to reconstruct 3D structures, while Szeliski [10] applies planar geometric constraints to improve camera pose estimation. Similarly, Dzitsiuk [11] employs planar surfaces for indoor 3D reconstructions, combining local plane fitting with global plane merging to effectively reduce reconstruction noise. These methods, however, are limited to scenarios containing three or more planes, restricting their applicability.

In small-scale indoor scenes, the weak texture characteristics of reconstructed objects often result in insufficient feature points, reducing reconstruction quality and accuracy [12]. Gao [13] proposed an adaptive stereo matching algorithm that integrates multi-dimensional information to improve feature-matching precision in weakly textured regions. However, while Gao’s method enhances matching accuracy, it reduces computational efficiency. Liu [14] used structured light scanning to acquire laser stripe image sequences for reconstructing low-height objects. This method is effective, but it is highly sensitive to lighting conditions. Zhang [15] employed fully convolutional neural networks for feature learning, addressing the challenges of reconstructing weakly textured surfaces. Although neural network-based methods offer robust feature learning, they tend to be more time-consuming compared to traditional approaches. Fiducial markers, commonly used in 3D reconstructions [16,17,18], provide high detectability and robust feature recognition, helping to mitigate challenges in low-texture scenarios. However, these methods suffer from pose ambiguity if marker corner detection is noisy. Jia [19] used artificial planar markers to enhance the robustness of camera pose estimation. The graph-filtering method in Jia’s approach resolves the pose ambiguity issues present in other marker-based methods [16,17,18], but it involves a more complex process for generating planar marker encodings. Zhang [20] proposed reconstructing objects by placing them on a printed newspaper to extract numerous feature points, compute camera intrinsic and extrinsic parameters, segment foreground contours, and generate voxels for reconstructions. While this method is effective in extracting feature points, it is limited by the quality of the printed newspaper and the complexity of the voxel generation process. Most of the methods based on plane-assisted labeling are used for SLAM (Simultaneous Localization And Mapping), and there are fewer methods used for SfM, and the plane markers used by previous authors are usually generated using special codes, which are more complicated. Therefore, in this paper, a black-and-white checkerboard grid calibration board is used as the planar marker, which is simpler to generate, while more constraints can be obtained. The details are as follows:

(1) A calibration scene based on a black-and-white chessboard is designed, where the corner points of the calibration board are known to lie on a single plane and exhibit multiple orthogonal relationships. Therefore, it can be used as a constraint during the camera pose estimation process. (2) Considering that in small-scale scenes, the size of the reconstructed objects is relatively small, making feature-point extraction and matching more difficult and hindering the effective acquisition of a sufficient number of high-quality matching point pairs, this paper designs a point-matching algorithm for texture-poor regions to enhance the feature points in an image.

## 2. Related Works

### 2.1. Structure from Motion

SfM is a photogrammetric technique that simultaneously recovers both camera poses and a 3D sparse point cloud of objects from a set of unordered images or video sequences. The early developments of SfM can be traced back to [21,22], where Snavely [23] built upon previous research to propose the Bundler pipeline, which was one of the first to successfully accomplish sparse 3D reconstructions from unordered images. VisualSFM [4], proposed by Wu as part of his research project, is a multi-core parallel SfM pipeline that accelerates the SIFT (Scale-Invariant Feature Transform) feature-extraction process through GPU acceleration. MVE [24], proposed by Simon Fuhrmann, is an SfM pipeline based on Bundler and VisualSFM, which combines both SIFT and SURF (Speeded Up Robust Features) feature-extraction methods, improving the completeness of the reconstructed point cloud. COLMAP [25], a more recent and advanced SfM pipeline, introduces a multi-modal geometric validation strategy that mitigates the drift effects often seen in incremental SfM, offering more robust reconstruction results. Some subsequent works [26,27,28,29,30] also enable the densification of the sparse point clouds output by SfM.

### 2.2. Feature Extraction and Matching

Feature-extraction algorithms primarily rely on keypoints in the image, which typically correspond to regions with significant gradient variations, such as corners, edges, lines, and spots. The FAST (Features From Accelerated Segment Test) [31] corner-detection algorithm determines whether a pixel is a corner by evaluating the difference between the pixel and its neighboring pixels. While FAST is fast, its limitations include the lack of scale and rotation invariance. The introduction of SIFT [32] addressed the issues of scale and rotation invariance in feature extraction. SIFT detects keypoints through scale-space extrema and uses local gradient-direction descriptors, providing both scale and rotation invariance. However, due to its multi-scale nature, SIFT is computationally expensive and relatively slow. SURF [33] improves upon SIFT by using the determinant of the Hessian matrix for feature-point detection and accelerates the computation using integral images, offering improved efficiency over SIFT. ORB (Oriented FAST and Rotated BRIEF) [34] combines FAST corner detection with binary feature descriptors, achieving lightweight and efficient performance. Additionally, methods like AKAZE (Accelerated-KAZE) [35] and BRISK (Binary Robust Invariant Scalable Keypoints) [36] have further explored robustness and efficiency in feature detection. Feature-matching techniques include methods such as brute force matching [37] and nearest neighbor matching [32], among others. Feature-point matching primarily involves techniques such as brute force matching and nearest neighbor matching. Brute force matching identifies the pair of points with the shortest distance between the left and right views as matching points, resulting in a higher miss-match rate. Nearest neighbor matching, on the other hand, selects the k-nearest features and computes the ratio of the distance between the closest point to that of the second closest. If this ratio is below a predefined threshold, the point pair is considered a valid match.

### 2.3. Planar Marker

In images with weak textures and significant viewpoint variations, feature-point extraction becomes challenging. Several methods utilizing planar markers [38,39,40,41] have been applied in structure from motion (SfM), where planar markers provide high-quality matching points for feature extraction, enabling more accurate pose estimation. These methods are widely used in 3D reconstructions. Although circular markers [42,43] offer advantages in detection under long-distance observation and visual blur, square planar markers [44,45,46,47] are preferred by researchers due to the ease of extracting their corner points. Furthermore, the use of planar markers can offer additional constraints for camera pose estimation, such as norm constraints and vertical vector constraints. However, planar markers typically consist of a wide black border, and a binary matrix inside that defines their identifier, making their creation process less convenient compared to black-and-white checkerboard calibration plates.

Therefore, this paper employs a black-and-white checkerboard calibration plate [48] as the planar marker to provide robust feature-point matching and additional planar constraints for the reconstruction.

## 3. Materials and Methods

**Figure 1:**
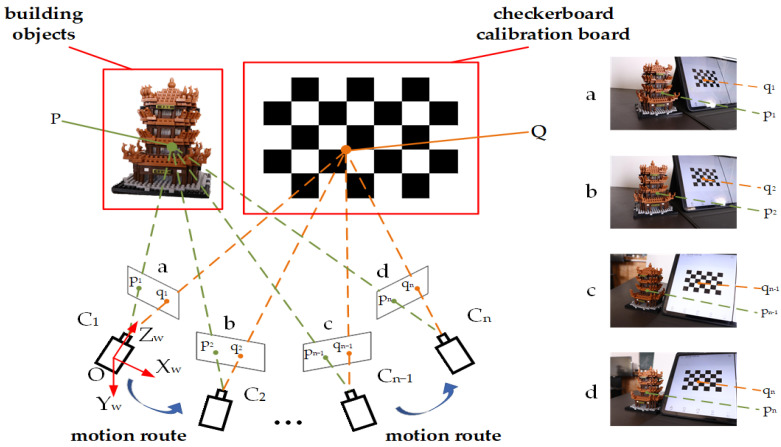
Diagram of auxiliary calibration scenario.

As shown in Figure 1, a black-and-white checkerboard calibration board is placed beside the object to be reconstructed, forming an auxiliary calibration scene. The world coordinate system is defined with its origin *O* at the center of the camera coordinate system of the first frame, and the axes *O*-X_W_Y_W_Z_W_ represent the world coordinate system. The camera’s pose is continuously adjusted during image acquisition, ensuring that the camera remains in front of the scene and maintains visibility of both the object and the calibration board throughout the process.

In this figure, points P and Q are visible from every viewpoint. The green dots represent feature points on the object, while the orange dots represent the corners of the calibration board, the green dashed line and the orange dashed line represent the propagation path of the camera’s line of sight, indicating that the camera is able to observe the object point P and the corner point Q. The blue arrows indicate the direction of the camera’s movement. *C*_1_, …*C_i_,* …, *C_n_* mark the camera positions during image acquisition, and a, b, c, and d represent some of the captured images.

### 3.1. Camera Pose Estimation During Reconstruction

SfM (structure from motion) is a commonly used method for 3D reconstructions, primarily applied to achieve 3D reconstructions from motion. During the reconstruction process, camera pose estimation begins by extracting feature points from the initial pair of images using SIFT. Subsequently, the essential matrix is computed based on the matched feature points. The essential matrix is then decomposed to obtain the camera poses, as shown in Equation (1).

(1) $$E=U\Sigma V^{T}$$

where **U** and **V** are orthogonal matrices and are diagonal matrices, usually. The singular value decomposition of the intrinsic matrix is obtained as follows:

(2) $$R_{1}=UWV^{T},R_{2}=UW^{T}V^{T},t=\pm u_{3},W=\left[\begin{array}{ccc} 0 & -1 & 0\\ 1 & 0 & 0\\ 0 & 0 & 1 \end{array}\right]$$

In Equation (2), **R**_1_ and **R**_2_ represent the possible rotation matrices, t is the potential translation vector, u_3_ is the third column of matrix **U**, and **W** is a constructed fixed matrix. There are four possible combinations of poses, and the correct pose combination is determined by triangulating the feature points and checking their re-projection errors.

**Figure 2:**
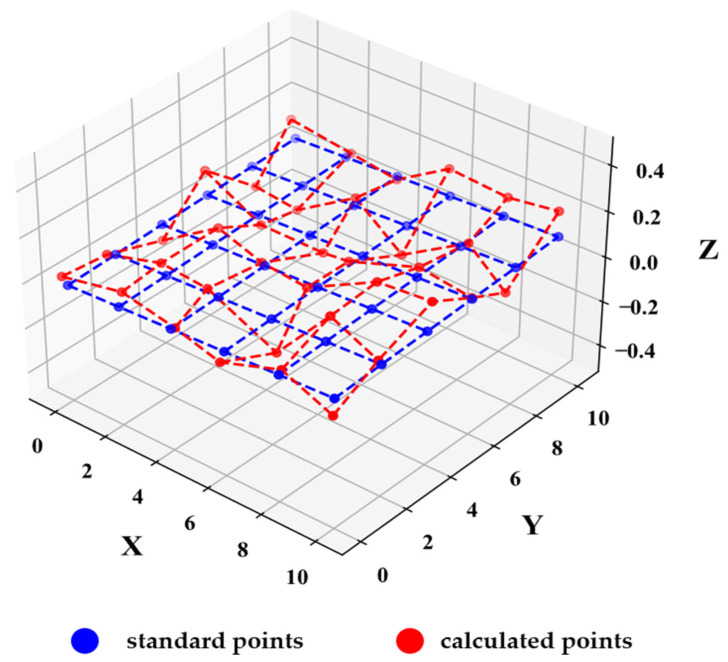
Diagram of corner positions difficult to meet the geometric characteristics of calibration board. (The red dashed line represents the irregular grid formed by connecting the red calculated points and the blue dashed line represents the regular planar grid formed by connecting the standard points).

After obtaining the initial camera pose, triangulation of the calibration board’s corner points is performed to calculate the 3D coordinates of each corner point. However, in real-world scenarios, image acquisition is often affected by noise, lens distortion, and other factors, making it difficult for the detected corners to fully satisfy the geometric properties of the calibration board. This primarily manifests as the corner points not lying on the same plane and the line segments formed by corner points along column directions and row directions not meeting orthogonality, as illustrated in Figure 2.

To mitigate the aforementioned issues, each corner point of the calibration board is constrained to a more accurate position. By utilizing the corner-point coordinates in conjunction with the plane equation, the constraints for the m corner points on the plane equation coefficients *a*, *b*, *c*, and *d* can be expressed as follows:

(3) $$AX=\left[\begin{array}{cccc} x_{0} & y_{0} & z_{0} & 1\\ \vdots & \vdots & \vdots & \vdots \\ x_{i} & y_{i} & z_{i} & 1\\ \vdots & \vdots & \vdots & \vdots \\ x_{m} & y_{m} & z_{m} & 1 \end{array}\right]{\left[\begin{array}{cccc} a & b & c & d \end{array}\right]}^{T}=0$$

When *m* > 4, the plane equation coefficients can be solved using the least squares method. After obtaining the plane equation, each corner point is projected onto the plane along the direction of the plane’s normal vector. The projection calculation for the corner point at the *i* row and *j* column is as follows:

(4) $$\mu_{ij}=\frac{ax_{ij}+by_{ij}+cz_{ij}+d}{a^{2}+b^{2}+c^{2}}$$

(5) $$P_{ij}^{\prime }=P_{ij}-\mu_{ij}n=(x_{ij}-\mu_{ij}a,y_{ij}-\mu_{ij}b,z_{ij}-\mu_{ij}c)$$

Here, P*_ij_* represents the coordinates of the corner point at the *i* row and *j* column, *μ_ij_* is the distance from the corner point at the *i* row and *j* column to the plane along the plane’s normal vector, and P*_i__,__j_*′ is the coordinates of the corner point after being projected onto the plane. The normal vector of the plane is denoted as **n** = (*a*,*b*,*c*).

**Figure 3:**
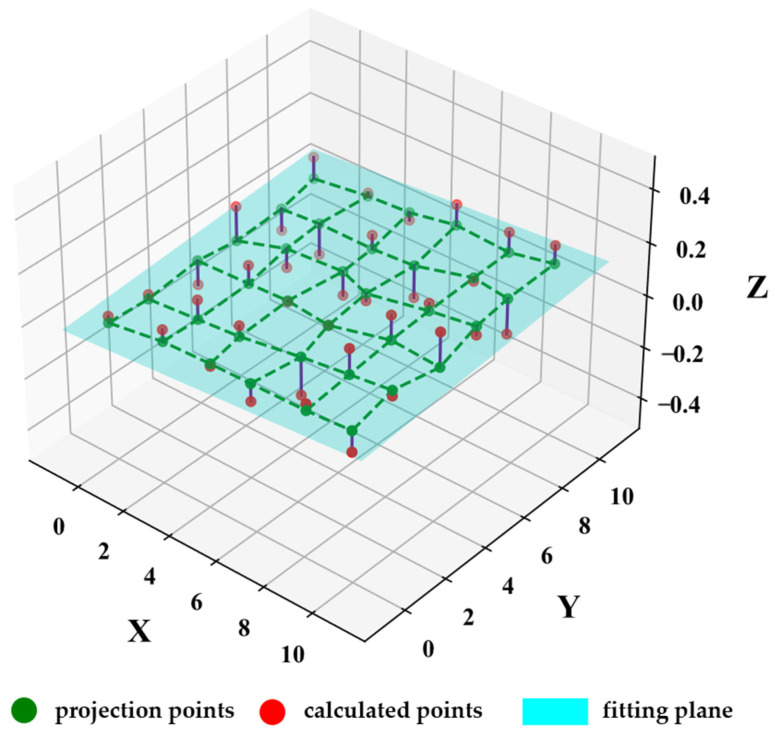
Diagram of corner projection onto plane still does not satisfy orthogonality. (The green dashed line indicates the irregular planar network formed by connecting the green projection points).

Even after constraining all corner points of the calibration board to the same plane, the orthogonality issue may still persist, as illustrated in Figure 3.

To obtain a more accurate camera pose through subsequent PnP solving, additional constraints are imposed on the calibration board’s corner points. This begins by fitting the line equations for each row and column of the calibration board corner points using the least squares method. The line equation fitting process for corner points in a single row is as follows:

(6) $$C_{i}=({\bar{x}}_{i},{\bar{y}}_{i},{\bar{z}}_{i})=\frac{1}{l}\sum_{i=0}^{l}P_{ij}^{\prime }(j=0,\ldots ,l)$$

(7) $$B=\left[\begin{array}{ccc} x_{0,j}^{\prime }-{\bar{x}}_{0} & y_{0,j}^{\prime }-{\bar{y}}_{0} & z_{0,j}^{\prime }-{\bar{z}}_{0}\\ \vdots & \vdots & \vdots \\ x_{i,j}^{\prime }-{\bar{x}}_{i} & y_{i,j}^{\prime }-{\bar{y}}_{i} & z_{i,j}^{\prime }-{\bar{z}}_{i} \end{array}\right]$$

(8) $$L_{i}(g)=C_{i}+gv_{max}$$

In this equation, **C***_i_* represents the centroid of the corner points in each row, **B** is the decentered matrix, **M** is the covariance matrix, and **L***_i_*(*g*) denotes the fitted line equation. **v***_max_* is the direction vector of the fitted line, obtained as the eigenvector corresponding to the largest eigenvalue from the eigenvalue decomposition of the covariance matrix **M**. *g* is a real-valued parameter. Similarly, the line equations for corner points in each column can be obtained.

Next, the direction vectors of the lines fitted for each row are sequentially dotted with those of the lines fitted for each column. The row and column line-direction vectors with the smallest dot product are selected as the constraint. Using the known length of the calibration board’s grid squares, each corner point is then constrained to an accurate position, as described below:

(9) $${\left\|p_{ij}^{\prime \prime }-p_{ij+1}^{\prime \prime }\right\|}_{2}=w,(i=0,\ldots ,r,\;j=0,\ldots ,l)$$

**Figure 4:**
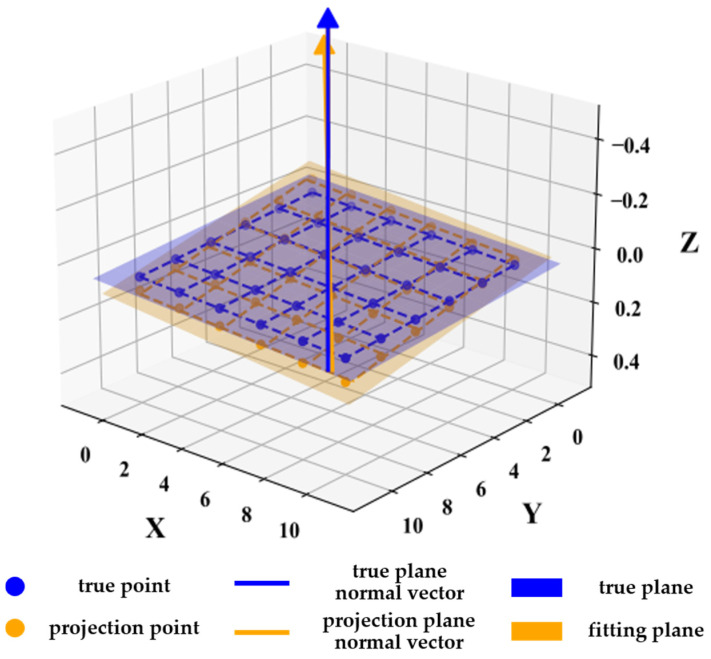
Diagram of the constrained corner position.

Here, point **P**′′ represents the corner point obtained after applying both the plane and line constraints. w denotes the known length of the calibration board grid squares, while *r* and *l* are the numbers of rows and columns, respectively. By applying these constraints, more accurate positions for the calibration board’s corner points can be determined, as illustrated in Figure 4.

After obtaining the 3D coordinates of all corner points, these corner points are matched with subsequent images using their sequences. The PnP (Perspective-n-Point) algorithm is then employed to estimate the camera poses of the additional images. Once the poses of all cameras are determined, a global BA (bundle adjustment) optimization is performed by minimizing the re-projection error, yielding more refined camera poses.

### 3.2. Feature-Point Enhancement Algorithm

In small-scale scenes, there may be issues with a limited number of feature points being extracted and matched, necessitating the enhancement of feature points. This process begins with an initial selection using epipolar geometry constraints, followed by further optimization with the Simpson error, and concludes with the selection of the best match based on re-projection errors. Specifically, we randomly generate a set of points in the weak texture regions of an image pair. Let the randomly generated points in the left-view image be denoted as *x_i_* = (*u_i_*, *v_i_*) and the corresponding points in the right-view image as *x_i_*′ = (*u_i_*′, *v_i_*′). Epipolar geometry constraints are constructed using the fundamental matrix **F**, where the points *x*_i_ and *x*_i_′ satisfy the following equation:

(10) $$x_{i}^{\prime T}Fx_{i}=0$$

By using Equation (11), an initial matching is performed to find points in the right view that satisfy the epipolar constraint with respect to the left-view points. Among these points, a stronger constraint, the Simpson error, is employed to filter out more accurate matches. The Simpson error is a measure that combines both geometric and pose information. For a given point pair *x_i_* and *x_i_*′, the Simpson error can be computed using the following formula:

(11) $$d\left(x_{i},x_{i}^{\prime }\right)=\frac{{\left(x_{i}^{\prime T}Fx_{i}\right)}^{2}}{{\left(Fx_{i}\right)}_{x}^{2}+{\left(Fx_{i}\right)}_{y}^{2}+{\left(x_{i}^{\prime T}F\right)}_{x}^{2}+{\left(x_{i}^{\prime T}F\right)}_{y}^{2}}$$

**Figure 5:**
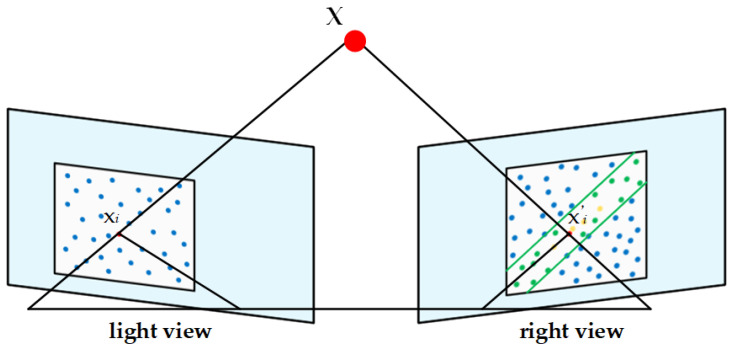
Comparison chart of a single transformation.

In the final matching point selection, we further optimize the process using re-projection errors. Since the camera poses for all views have been determined through the calibration board corner points, we use the known camera poses to triangulate the point pairs and to compute the re-projection error sequentially. The point pairs with the smallest re-projection error are selected as the final matches, as shown in Figure 5, where blue dots denote generated random points, green parallel lines denote the range in which point *x_i_* in the left view may find a matching point in the right view, yellow dots denote match able random points in this range, *x_i_*’ denotes the point with the smallest reprojection error, and **X** denotes the spatial three-dimensional point corresponding to *x_i_* in the left view and *x_i_*′ in the right view.

### 3.3. Sparse Reconstruction

**Figure 6:**
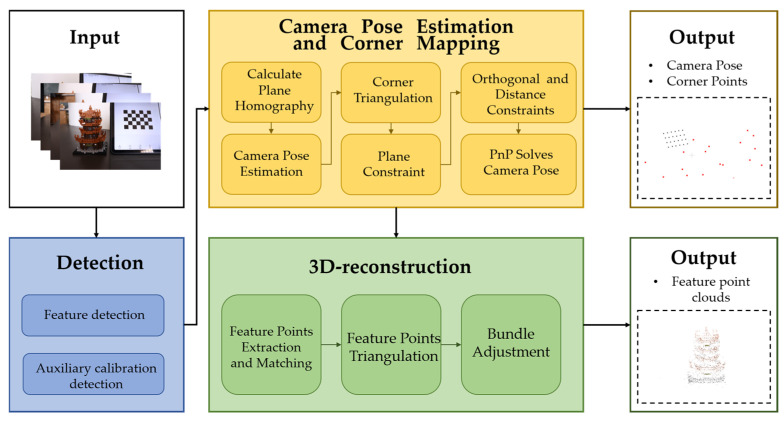
3D reconstruction flowchart under auxiliary calibration field. (The red points are the camera position, the black points are the calibration plate corner points, and the brown points are the point clouds of the reconstructed object).

In the aforementioned process, the camera poses for all captured images and enhanced feature-point matches are obtained. The two views with the most feature-point matches are selected for the initial reconstruction. Since the images were captured sequentially, the images are added to the initial reconstruction in the same order, which reduces computational complexity while ensuring the reliability of the matches. Given that the camera poses have already been determined using the calibration board, bundle adjustment (BA) optimization is not performed after each image addition. Instead, global BA optimization is conducted after all images have been processed to ensure the accuracy of the entire 3D reconstruction model, as shown in Figure 6.

## 4. Results

**Figure 7:**
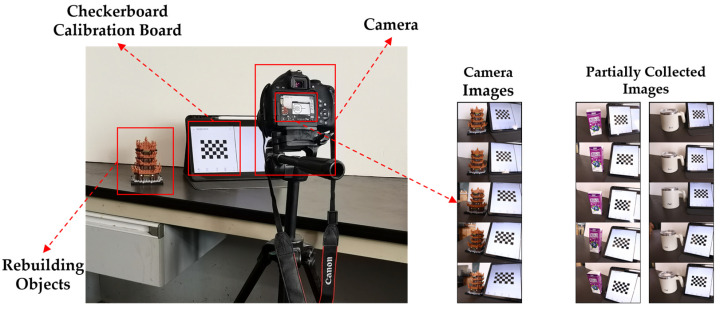
Diagram of experiment acquisition of image patterns. (The three sets of images depict brown-colored blocks resembling the Yellow Crane Tower, purple-colored Chinese brand yogurt drink and a white-colored water cup).

To validate the effectiveness of the proposed method in real-world scenarios, a monocular camera was used to capture images of the scene and to perform the reconstruction. The image acquisition setup is shown in Figure 7. The experimental camera is a Canon 800D DSLR with a 24.2-megapixel sensor, featuring a 22.3 × 14.9 mm CMOS sensor and a DIGIC image processor. Considering that complex backgrounds can introduce noise during the reconstruction process, a white foam board was placed in the scene after auxiliary calibration to reduce such interference. The images were captured with the object to be reconstructed and the auxiliary calibration scene, both visible, ensuring controlled shooting conditions.

Before the reconstruction experiment, camera-intrinsic parameters need to be calibrated. Due to the reconfiguration of the scene, the captured images contain a black-and-white checkerboard calibration board. Therefore, there is no need to capture additional images for camera calibration; the scene images themselves are directly used to calibrate the camera’s intrinsic parameters. The resulting camera-intrinsic matrix is as follows:

$$K=\left[\begin{array}{ccc} 4937.668 & 0 & 3000\\ 0 & 4937.668 & 2000\\ 0 & 0 & 1 \end{array}\right]$$

### 4.1. Analysis of Factors Influencing Calibration Board

To solve for the exterior orientation parameters using corner points in the subsequent reconstruction process, it is crucial to ensure the accuracy of the initial point cloud from the two images. Therefore, this study analyzes the impact of the number of calibration board corners, the size of the checkerboard squares, and the shooting distance on the accuracy of the reconstructed corners. In the experimental analysis, checkerboard images with grid square sizes of 10 mm, 13 mm, 16 mm, and 19 mm and corner-point configurations of 3 × 5, 4 × 6, and 5 × 7 were generated. The images were captured from distances of 50 cm and 75 cm in front of the scene. The re-projection error of the corner reconstruction results was used as the standard to evaluate accuracy: a smaller re-projection error indicates higher accuracy. The average re-projection errors for different checkerboard sizes are shown in Table 1:

From the results in Table 1, it can be observed that the average re-projection error is smallest when the checkerboard grid square size is 16 mm, and the shooting distance is 50 cm. For checkerboards with grid sizes of 10 × 10 mm and 13 × 13 mm, the re-projection error is relatively large. This is because smaller calibration boards have fewer pixel areas for the corner points in the image, making them more susceptible to noise interference, which leads to errors during corner-point extraction. On the other hand, larger calibration boards can also cause detection issues, as the corner points occupy a larger pixel area and may not be precisely located at the exact pixel position, thus affecting accuracy. This explains why the reconstruction results for the 19 × 19 mm checkerboard are suboptimal. Additionally, due to camera distortion, the larger the calibration board, the more it is affected by distortion in the image.

Furthermore, the re-projection errors for images captured at a 50 cm distance from the scene are consistently smaller than those captured at a 75 cm distance. This is primarily because, as the distance increases, the disparity decreases, which reduces image details. For checkerboards with different numbers of corner points, the re-projection error differences are minimal, indicating that the number of corner points has a minor impact on accuracy.

Therefore, a 4 × 6 corner configuration with a checkerboard grid square size of 16 mm is selected to construct the scene, and images are captured at a distance of 50 cm from the scene.

### 4.2. Comparing Feature Point Extraction Algorithms on the Local Dataset

**Figure 8:**
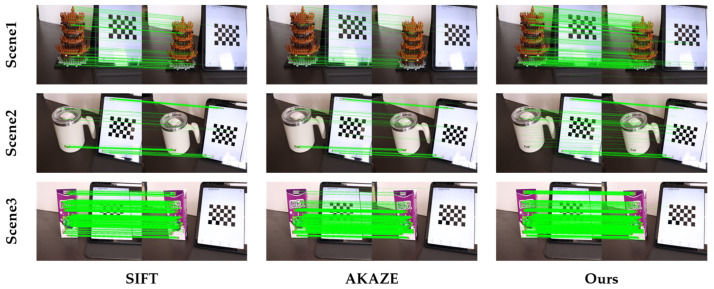
Comparison diagram of feature-point extraction and matching of initial image pairs. (The green line represents the line between the correctly matched points).

This study performs registration experiments on three sets of captured images using SIFT, AKAZE, and the feature-point extraction algorithm proposed in this paper. The matching results of the initial image pairs in the three datasets are shown in Figure 8. The performance comparison of the three algorithms is presented in Table 2. The comparison analysis uses the correct matching number (CMN), re-projection error, and running time as quantitative evaluation criteria for registration accuracy.

**Figure 9:**
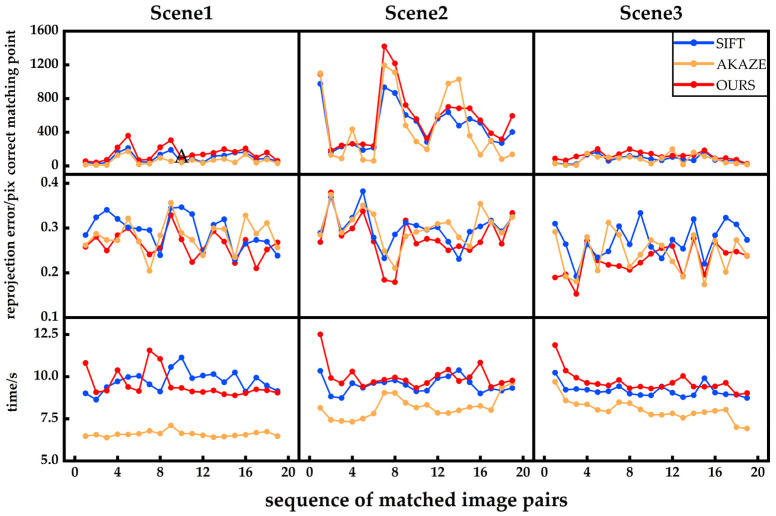
Comparison diagram of feature-point extraction and matching.

Figure 8 demonstrates the feature-point extraction and matching results of SIFT, AKAZE, and the proposed algorithm on the initial image pairs for the reconstruction. As shown, the proposed algorithm significantly increases the number of correctly matched point pairs. Moreover, from Table 2, it is evident that, compared to SIFT and AKAZE, the proposed method not only increases the number of matched points but also slightly improves accuracy and remains at the same time-complexity level as SIFT. For the third set of captured images, the number of correctly matched feature points increased by 486 and 225, respectively, while the re-projection error was reduced by 0.0482 and 0.0644 pixels, and the running time also increased by only 0.4 s compared to SIFT, respectively, proving that the proposed method improves the accuracy of the initial reconstruction and does not introduce excessive computational complexity. The correct matching number, re-projection error, and running time for all image pairs are shown in Figure 9.

From Figure 9, it can be observed that the proposed method increases the number of correct matches. Since the proposed method is based on SIFT for feature-point extraction, it extracts more feature points compared to SIFT. However, AKAZE, which uses a nonlinear diffusion filter, smooths the image and suppresses noise and less prominent features in weak texture areas. This results in fewer extracted feature points in the first and third datasets when compared to SIFT and the proposed method. Likewise, because the proposed algorithm is an improvement over SIFT, and although the running time increases a little compared to SIFT in most cases, it is still within the same order of magnitude. It yields a lower re-projection error compared to SIFT. Therefore, compared to other methods, the proposed algorithm enhances the accuracy of matching, providing better matching pairs for reconstructions and does not significantly increase the computational complexity compared to SIFT.

### 4.3. Evaluation of Feature Point Extraction Algorithms on the ETH3D Dataset

**Figure 10:**
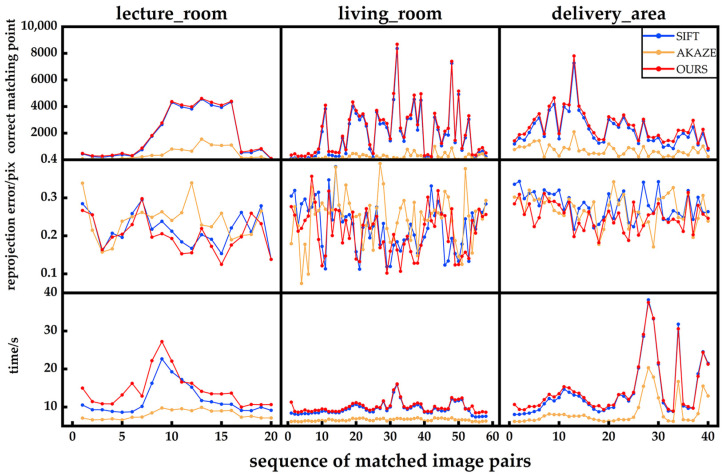
Comparison of feature-point extraction and matching for ETH3D dataset.

**Figure 11:**
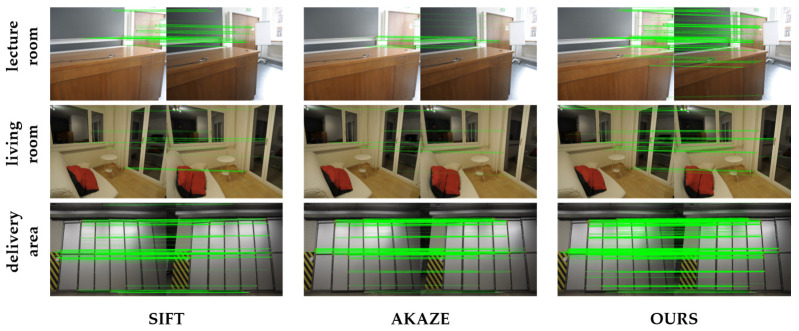
Comparison of feature-point extraction and matching for some images in ETH3D dataset. (The green line represents the line between the correctly matched points).

**Figure 12:**
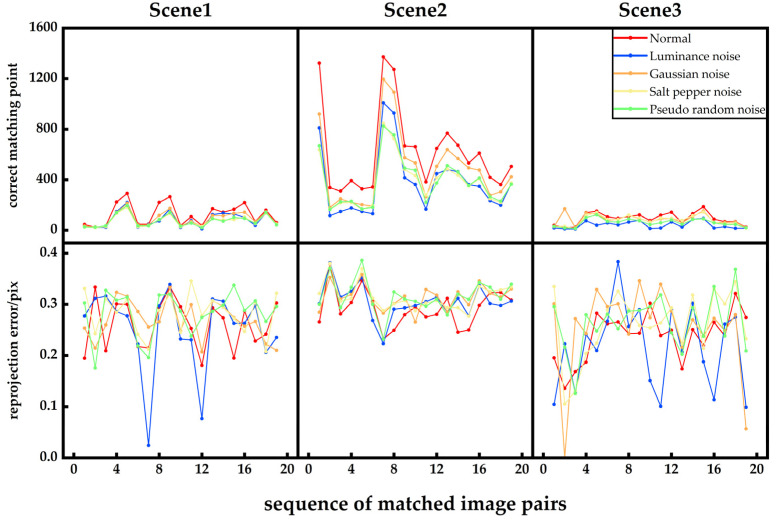
Comparison of different noise feature-point extraction and matching.

To validate the robustness of the feature-point enhancement method proposed in this paper, two sets of experiments were conducted. The first experiment aimed to assess the adaptability of the method under different environmental conditions. Three indoor image sets from the public ETH3D dataset were selected for a comparison of feature-point algorithms, namely, lecture_room, living_room, and delivery_area. In this experiment, we compared the performance of SIFT, AKAZE, and the proposed method in terms of correct matching pairs, re-projection errors, and runtimes. The experimental results are presented in Figure 10, with some visualizations of the image-matching results shown in Figure 11. The second experiment was conducted on a local image dataset, where feature-point extraction and matching were performed under different types of noise interference. Specifically, Gaussian noise, salt-and-pepper noise, pseudo-random noise, and varying light contrasts were introduced to simulate the disturbances that images may encounter in various environments. The experiment compared the correct matching pairs and re-projection errors between the noisy images and the normal images. Runtime comparisons were not performed, as both use the same feature-point extraction and matching algorithm methods. The results are shown in Figure 12.

As shown in Figure 10, since the proposed method is an improvement upon SIFT, its curves for correct matching pairs and runtimes exhibit similar trends to those of SIFT. However, the proposed method consistently demonstrates a slightly higher number of correct matching points and, in most cases, yields lower re-projection errors compared to SIFT, indicating stronger adaptability to different environments. Figure 11 shows that, compared to SIFT and AKAZE, the proposed method increases the number of successfully matched point pairs. The results in Figure 12 indicate that although the number of correct matching points decreases slightly under noise interference, it remains within an acceptable range, demonstrating the robustness of the proposed algorithm to noise.

### 4.4. Sparse-Point-Cloud Reconstruction

After obtaining the initial camera poses and the point cloud of the calibration board corner points, the corner points of the calibration board are directly matched with the corner points in the remaining images. Then, the PnP algorithm is used to solve for the poses of all cameras, followed by a global bundle adjustment (BA) optimization to obtain the optimal camera poses for all images. For object reconstructions, since the images were captured sequentially, the image-addition strategy is to directly add the images in the sequence.

**Figure 13:**
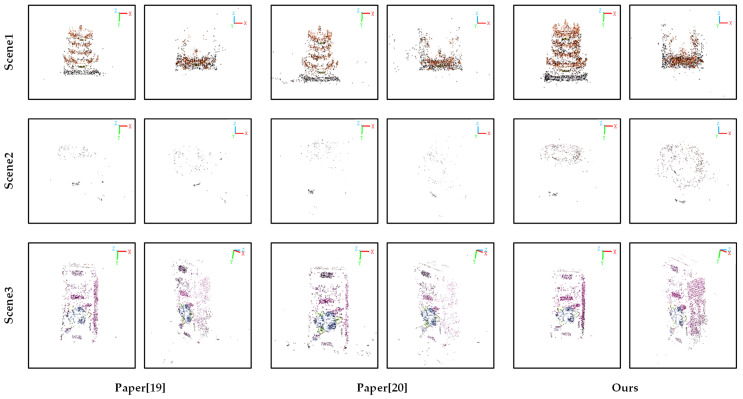
Sparse-point-cloud comparison image. (The brown point cloud represents the reconstruction result of the Yellow Crane Tower blocks, the beige point cloud represents the reconstruction result of the water cup, and the purple point cloud represents the reconstruction result of the yogurt drink carton).

The proposed method is compared with the methods in [19] and [20], using the number of points in the point cloud, re-projection errors, and running times as the evaluation criteria for reconstruction accuracy. The reconstruction results are shown in Figure 13, and the evaluation data are presented in Table 3.

As shown in Figure 13, for any given set of captured images, the proposed method yields a more complete and denser point cloud compared to the methods in [19,20], providing a stronger visualization. From Table 3, it can be seen that the proposed method achieves higher accuracy across all three experimental datasets. Specifically, the proposed method has the smallest average re-projection error of 0.4151 pixels. In this dataset, compared to the methods in [19,20], the average re-projection errors are reduced by 0.2185 pixels and 0.1945 pixels, respectively. For the other datasets, the proposed method also reduces the average re-projection error, demonstrating that the proposed method offers higher accuracy compared to the alternative methods. However, due to the extra step of calibrated-plate corner-point extraction required by the method in this paper and the extra slight computational complexity introduced by the feature-point enhancement method, the running time is not the shortest, is second to [19], and is slightly better than [20].

## 5. Conclusions

This paper proposes an auxiliary calibration scene and leverages this calibration along with the SfM (structure from motion) method for 3D object reconstructions, addressing the issues of sparse features and inaccurate pose estimation in small-scale scene reconstructions. The proposed method reliably extracts a sufficient number of calibration board corner points and uses these points to estimate the camera pose. In the subsequent image-addition and reconstruction process, the corner-point sequence can be directly used for matching, enabling a more efficient, accurate, and convenient estimation of camera-exterior orientation parameters. Additionally, plane, orthogonality, and distance constraints are incorporated during the camera pose estimation process, enhancing the accuracy of the camera pose estimation.

Furthermore, this study improves the feature-point extraction and matching algorithms, resulting in more correct matches and does not add too much computational complexity. From the final reconstruction results, it can be observed that due to scene limitations, the proposed method is unable to reconstruct certain parts of the 3D model beyond the auxiliary calibration scene. Future research will focus on improving this aspect to enhance the overall reconstruction and visual quality.

## Figures and Tables

**Table 1:** Comparison results of re-projection errors for chessboard grids of the same size.

Shooting Distance/cm	Chessboard Grid Edge Length/mm	Errors/pix			
		Scene1	Scene2	Scene3	Average
50	10	1.7967	1.7979	1.7982	1.7976
50	13	0.4735	0.4763	0.4767	0.4755
50	16	0.2461	0.2453	0.2495	0.2470
50	19	0.7483	0.7534	0.7569	0.7528
75	10	1.9869	1.9827	1.9785	1.9827
75	13	0.5763	0.5726	0.5803	0.5764
75	16	0.3833	0.3820	0.3865	0.3840
75	19	0.8059	0.8037	0.7942	0.8012

**Table 2:** Comparison results of feature extraction and matching of initial image pairs.

Scenes	SIFT			AKAZE			Ours		
	CMN	Re-projection Error/pix	Time/s	CMN	Re-projection Error/pix	Time/s	CMN	Re-projection Error/pix	Time/s
Scene1	190	0.3445	9.4221	55	0.3389	7.7324	304	0.3389	9.3913
Scene2	118	0.2633	10.1486	106	0.2137	6.4070	198	0.2063	9.1821
Scene3	933	0.2323	10.0145	1194	0.2485	7.8439	1419	0.1841	10.4204

**Table 3:** Comparison of sparse-point-cloud reconstruction results.

Scenes	Methods	Points Number	Re-projection Error/pix	Time/s
Scene1	Paper [19]	2216	0.7079	93.5579
	Paper [20]	2680	0.6402	137.6154
	Ours	4538	0.5245	123.2882
Scene2	Paper [19]	634	0.6459	97.8644
	Paper [20]	439	0.6493	104.6297
	Ours	822	0.4996	100.1918
Scene3	Paper [19]	4917	0.6336	95.0657
	Paper [20]	4609	0.6096	113.6048
	Ours	4925	0.4151	102.6768

## Data Availability

The original contributions presented in the study are included in the article; further inquiries can be directed to the corresponding author.
